# Genome-Wide Analysis of BURP Domain-Containing Gene Family in *Solanum lycopersicum* and Functional Analysis of *SlRD1* Under Drought and Salt Stresses

**DOI:** 10.3390/ijms252312539

**Published:** 2024-11-22

**Authors:** Huiru Sun, Jinyu Yang, Bei Fan, Min Ren, Yanfeng Wang, Guoliang Chen, Guoting Cheng

**Affiliations:** 1 College of Life Sciences, Yan’an University, Yan’an 716000, China; yangjinyu202410@163.com (J.Y.); 15596191125@163.com (B.F.); minren_edu@163.com (M.R.); yadxwyf@yau.edu.cn (Y.W.); glc9359@163.com (G.C.); chengguoting0417@yau.edu.cn (G.C.); 2 Shaanxi Key Laboratory of Research and Utilization of Resource Plants on the Loess Plateau, College of Life Sciences, Yan’an University, Yan’an 716000, China

**Keywords:** BURP domain-containing genes, tomato, drought, salt, *SlRD1*

## Abstract

The BURP domain-containing (*BURP*) genes belong to plant-specific families and are known as essential for various biological processes in plants. However, knowledge of the functions of *BURP* genes in tomato (*Solanum lycopersicum*) is lacking. In our study, bioinformatics analysis was performed for the *SlBURP* gene family, including phylogeny, chromosomal localization, gene structure, *cis*-acting elements and expression. In addition, the function of *SlRD1* in drought and salt stresses was explored. In tomato, fourteen *BURP* family members were identified, located on five chromosomes, including two tandem duplication clusters. These BURP members were classified into four subfamilies. The promoter regions of *SlBURPs* harbored numerous hormone- and stress-response elements. Tissue expression analysis showed that several *SlBURPs* were highly expressed in roots, flowers or fruits. Meanwhile, the expressions of most *SlBURPs* could be regulated by drought, salt and cold treatments, and some of them also responded to ABA treatment. Moreover, the ectopic expression of *SlRD1* in *Arabidopsis* enhanced tolerances to drought and salt stresses and increased the sensitivity of seed germination to ABA. In conclusion, the comprehensive analysis of the *SlBURP* family in tomato and the functional exploration of *SlRD1* in drought and salt stresses provide a basis for further dissecting the roles of tomato *BURP* genes.

## 1. Introduction

Tomato (*Solanum lycopersicum* L.) is a crucial vegetable crop and is cultivated worldwide [1]. However, various environmental stresses severely impact the yield and quality of tomato during its development [2]. Plants have evolved complex networks to respond to different environmental challenges. The *BURP* domain-containing (BURP) proteins have shown important functions in plant development and stress resistances [3,4,5]. Therefore, the study of tomato *BURP* genes could provide a theoretical basis for a better understanding of the stress response mechanism of tomato. The main feature of *BURP* proteins is the conserved *BURP* domain in the C-terminus, which included four repeated cysteine-histidine (CH) and several highly conserved amino acids. The *BURP* family’s name is based on the four protein members (BNM2, USP, RD22 and PG1β) previously discovered [6].

To date, the *BURP* gene family has been studied in numerous plants, including alfalfa (*Medicago truncatula*) [7], jujube (*Ziziphus jujuba*) [8], cotton (*Gossypium hirsutum*) [6], common bean (*Phaseolus vulgaris*) [9] and rose (*Rosa chinensis*) [10]. The BNM2-like, USP-like, RD22-like and PG1β-like subfamilies were four classic subfamilies of *BURP* family [10]. In addition, from further studies, other subfamilies were identified in the *BURP* family [6]. Studies on the functions of some *BURP* genes showed diverse roles during different processes of plant growth. For example, BNM2 (a BURP protein of *Brassica napus*), VfUSP (a BURP protein of *Vicia faba*) and ASG1 (a BURP protein of *Panicum maximum*) all might regulate spore formation during embryonic development [11,12,13]. OsRAFTIN1 and RA8 (two BURP proteins of rice) were involved in another development [14,15]. *AtUSPL1*, a member of the RD22-like subfamily, was involved in seed development [16]. Additionally, *SCB1* (a *BURP* gene of soybean) was expressed specifically in the seed coat and might have an important role in the cell differentiation of the seed coat [17]. *GhRD1*, a *BURP* gene in cotton, activated by GhHOX3 and GhHD1, promoted cotton fiber elongation [18]. PG1β, a BURP protein in tomato, was involved in fruit maturation, as it affects pectin solubilization and degradation [19,20].

Moreover, many *BURP* genes, especially the members of the RD22-like subfamily, have been found to be involved in stress response. *AtRD22* played a regulatory role in the drought stress response as an inhibitor and was also induced by abscisic acid (ABA) treatment [5]. *MdRD22* could be activated by MdWRKY115 and enhances the resistance to drought and osmotic stress [21]. The overexpression of *GmRD22* in *Arabidopsis* and rice could improve the tolerance to salt stress, possibly through increasing lignin production [22]. The expressions of *BgBDC1*, *2*, *3* and *4*, four *BURP* members of the RD22-like subfamily in the mangrove (*Bruguiera gymnorrhiza*), could be regulated by different abiotic stress and ABA treatments [23]. The overexpression of *OsBURP16* could enhance the sensitivity of transgenic rice to various abiotic stresses through affecting pectin content of the cell wall [4]. The ectopic expression of *PpBURP2* in rice could enhance the tolerance of plants to salt and drought stresses [3]. The ectopically expressed *Sali3-2* in *Arabidopsis* seedings showed a higher tolerance to metal ions (cadmium and copper) [24]. These findings suggested that *BURP* genes, especially the member of the RD22-like subfamily, might also show important potential functions during tomato response to stress. Currently, the roles of *BURP* genes in many plants have been studied, with research focusing on plant development and stress responses. However, little has been reported about the functions of tomato *BURP* genes, especially in response to stress, which has limited our comprehension of tomato *BURP* family and resulted in a lack of knowledge on the roles of *SlBURPs*.

In order to better understand the potential roles of tomato *BURP* genes, a systematic analysis of *BURP* genes in tomato was performed, including gene structure, conserved motifs, phylogenic tree, chromosomal localization, *cis*-acting elements and expressions in various tomato tissues, under abiotic stress and ABA treatments. In the future, the tomato *BURP* genes could be chosen or used to deeply study according to the above results. Above all, the ectopic expression of *SlRD1*, a member of the RD22-like subfamily, enhanced the salt tolerance of transgenic *Arabidopsis* plants and improved ABA sensitivity of transgenic *Arabidopsis* seeds. The results demonstrated the function of *SlRD1* in response to abiotic stresses and the association with ABA signaling pathway. These data are expected to provide a basis for further research on the functions of tomato *BURP* genes.

## 2. Results

### 2.1. Identification of BURP Gene in Tomato

The 14 BURP members (Table S1) in the tomato genome were identified via HMMER (Hidden Markov Model) screening with the BURP domain model file (PF03181), verified on the SMART website and named based on their homology with BURP members of *Arabidopsis* and other reported species. The length range of SlBURP proteins was from 132aa (SlUSP1) to 636aa (SlPG4), and their molecular weight ranged from 14.76 kDa to 69.47 kDa. The range of their isoelectric point values was from 5.13 to 9.71. The grand average of hydropathicity indicated that all SlBURP proteins, except SlPG5, were hydrophilic (Table S1).

### 2.2. Phylogenetic Analysis and Chromosomal Location of SlBURPs

**Figure 1:**
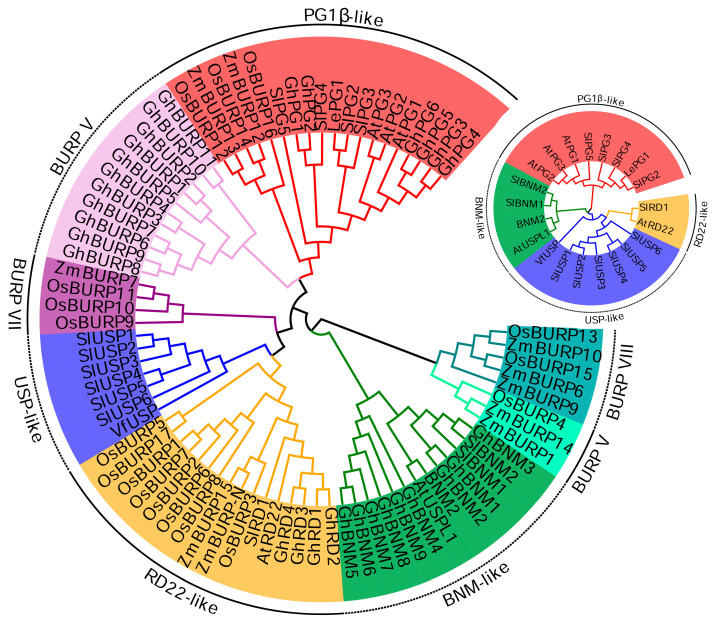
Phylogenetic trees of BURP proteins in different species. *Arabidopsis thaliana*, *Oryza sativa*, *Zea mays*, *Gossypium hirsutum*, *Vicia faba* and *Solanum lycopersicum* are labeled as At, Os, Zm, Gh, Vf and Sl, respectively. The inserted phylogenetic tree was constructed using BURP proteins from tomato and *Arabidopsis*. Distinct color sections represent eight subfamilies.

In order to explore the phylogenetic relationships of SlBURP family members, 78 BURP proteins from tomato (14), *Arabidopsis* (5), rice (17), maize (10) and cotton (30) and two host BURP proteins (BNM2 and VfUSP) were used to construct the phylogenetic tree (Figure 1). The eight subfamilies, including four classic subfamilies, BURP V, BURP VI, BURP VII and BURP VIII were divided, which comprised BURP members from 1 (USP-like and BURP V subfamilies) to 5 (PG1β-like subfamily) species. Additionally, the PG1β-like and BURP VI subfamilies contained the most (19) and the fewest (3) BURP members, respectively. The BURP family members in tomato were distributed into BNM2-like, USP-like, RD22-like and PG1β-like subfamilies (Figure 1).

**Figure 2:**
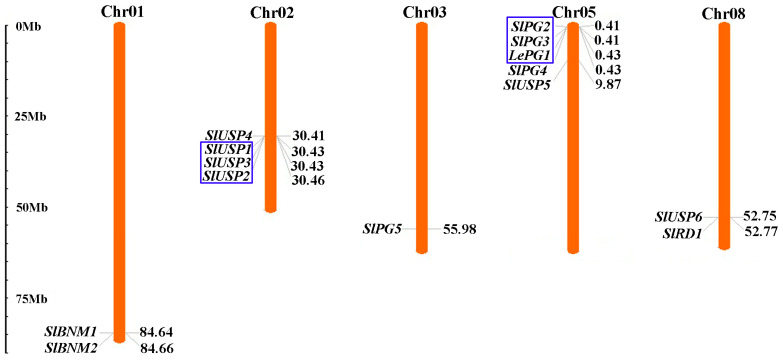
Chromosomal location of *SlBURPs*. The blue boxes represent tandem duplicated genes.

The distribution of *SlBURPs* on tomato chromosomes showed that these genes were unevenly located on five chromosomes (Figure 2). Among them, chromosome 05 contained the most *SlBURPs* (5), followed by chromosome 02 (4). Chromosomes 01 and 08 contained two *SlBURPs*, while chromosome 03 only contained one *SlBURP* (*SlPG5*). The analysis of duplication events in the *SlBURP* family showed two tandem duplication clusters, containing three (*SlUSP1* to *SlUSP3*, and *LePG1* to *SlPG3*) *SlBURPs*, respectively. The results indicated that the expansion of USP-like and PG1β-like subfamilies in tomato may derive from tandem duplication events.

### 2.3. Gene Structure and Conserved Motif Analysis of SlBURPs

**Figure 3:**
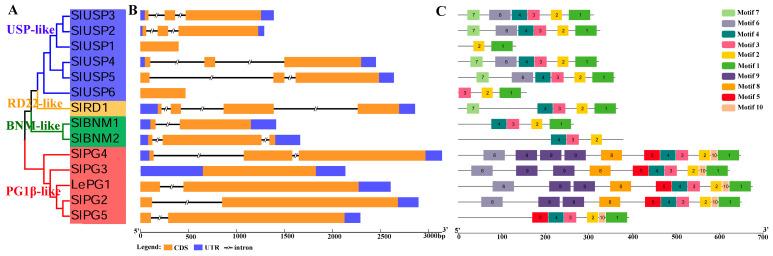
Phylogenetic tree (**A**), gene structure (**B**) and conserved motif (**C**) analysis of *SlBURP* family.

The analyses of the intron exon structure and conserved motifs were performed to explore the conservation and diversification of tomato *BURP* family genes (Figure 3, Table S1). The results showed that six *SlBURPs* contained three exons and two introns, four *SlBURPs* contained two exons and one intron, three *SlBURPs* contained one exon, and *SlRD1* contained four exons and three introns (Figure 3B and Table S1). The conserved domain analysis showed that all SlBURP proteins contained BURP domain, with half of them also containing a signal peptide (Figure S1A, Table S1). The conserved amino acid sites in the BURP domain were further analyzed using a multiple sequence alignment. The four repeat CH motifs in SlBURPs were summarized as CH-X_10_-CH-X_25-27_-CH-X_25_-CH-X_8_-W (Figure S1B).

In addition, the 10 conserved motifs in SlBURPs were further investigated (Figure 3C). The motifs in the same subfamily showed similar numbers and distributions. Among them, motif 2, motifs 1 and 3, and motif 4 were present in 14, 13 and 12 SlBURPs, respectively. Motifs 5, 8, 9 and 10 were only present in the PG1β-like subfamily. Motif 6 was present in the USP-like and PG1β-like subfamilies. Motif 7 was present in the USP-like and RD22-like subfamilies. Overall, these results revealed that closely related SlBURPs had more similar gene structures and motif arrangements.

### 2.4. Cis-Acting Element Analysis in Putative SlBURP Promoters

**Figure 4:**
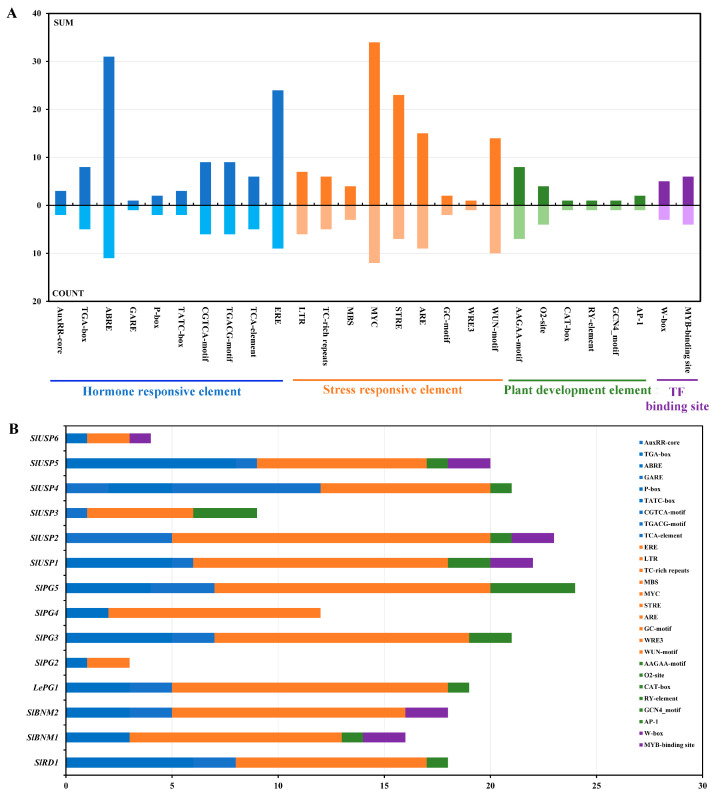
Analysis of *cis*-acting elements in *SlBURP* promoters. (**A**) The statistics of different *cis*-acting elements in the promoters of *SlBURP* family genes. (**B**) The distribution of different *cis*-acting elements in *SlBURP* promoters. SUM: the total number of *cis*-acting elements in *SlBURP* promoters; COUNT: the number of *SlBURPs* whose promoter contains the respective elements; different colors represent different element types.

We analyzed the *cis*-acting elements in the promoters of *SlBURPs* to explore the potential roles of *SlBURPs* (Figure 4). Four types of elements, including hormone response, stress response, plant development and transcription factor binding elements, were investigated. The hormone response elements included response to ABA (AREB), ethylene (ERE), auxin (AuxRR-core and TGA-box), gibberellin (GARE, P-box and TATC-box), methyl jasmonate (CGTCA-motif and TGACG-motif) and salicylic acid (TCA-element) elements. Notably, ABA- and ethylene-responsive elements were widely distributed in *SlBURP* promoters, with 11 and 9, respectively. The stress response elements, especially MYC, WUN-motif and ARE, were ubiquitously present in all *SlBURP* promoters. The plant development elements were present in 10 *SlBURP* promoters. Among them, the AAGAA-motif, involved in secondary xylem development, was most widespread, being present in seven *SlBURP* promoters. In addition, binding sites for the WRKY and MYB transcription factors were present in several *SlBURP* promoters, indicating that these *SlBURPs* may be regulated by upstream transcription factors. These results indicated that *SlBURPs* may be involved in abiotic stress response and hormone response.

### 2.5. Tissue Expression of SlBURPs

**Figure 5:**
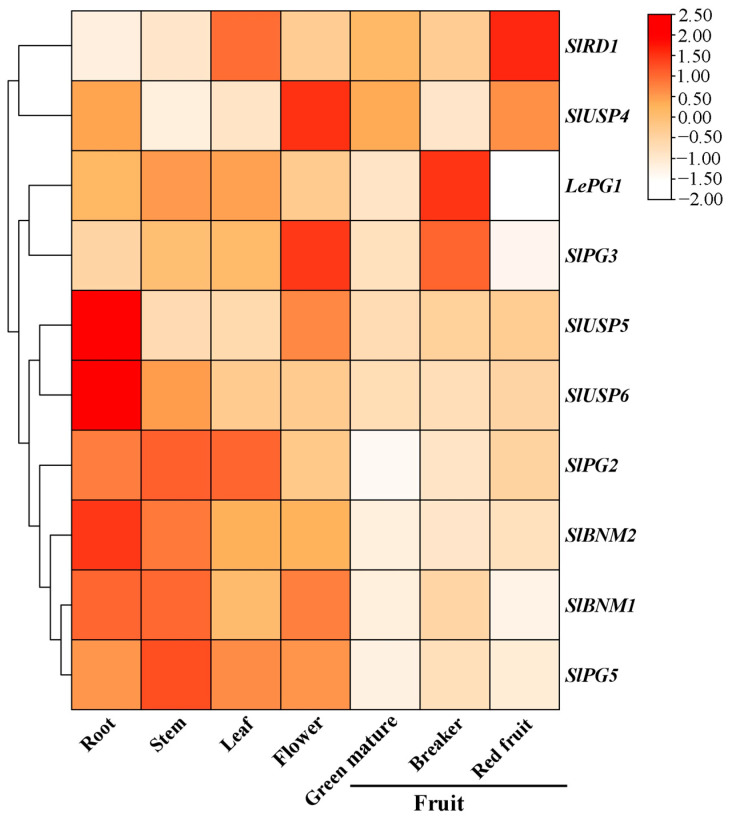
Expressions of *SlBURPs* in different tomato tissues. The numerical values on the right represent the log_2_ normalization of qRT-PCR values.

To explore the potential functions of *SlBURPs* during tomato growth, qRT-PCR was used to analyze the expression of 10 *SlBURPs* (excluding *SlUSP1*-*SlUSP3* for high sequence similarity and *SlPG4* for almost no expression in different tissues) in various tomato tissues, including roots, stems, leaves, flowers and fruits at different stages (Figure 5). The expressions of *SlBURPs* showed spatio-temporal specificity. *SlPG2*, *SlBNM1*, *SlBNM2* and *SlPG5* were highly expressed in different tissues, except fruits, at different stages. Notably, *LePG1* and *SlRD1* showed generally high expression in different tissues, especially in breaker stage or red fruit. Additionally, *SlUSP5* and *SlUSP6* exhibited root specific expressions, and *SlUSP4* and *SlPG3* were highly expressed in flowers. The results indicate that *SlBURPs* may perform various functions in plant development.

### 2.6. Expression Analysis of SlBURPs Under Abiotic Treatments

**Figure 6:**
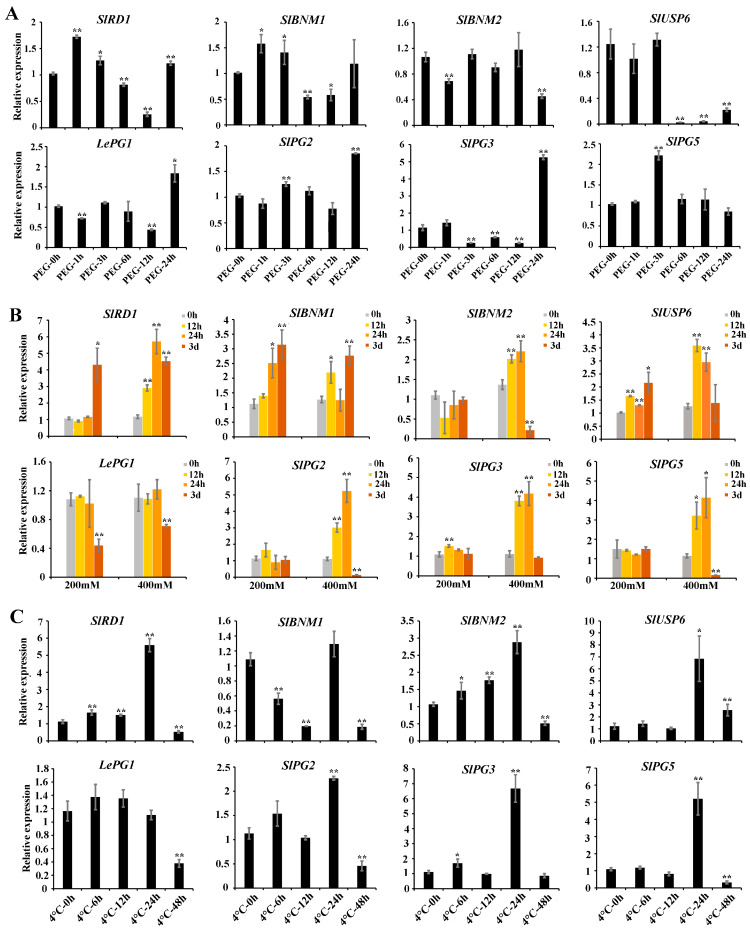
Expressions of 8 *SlBURPs* in tomato leaves after drought (**A**), salt (200 mM and 400 mM) (**B**) and cold (4 °C) treatments (**C**). The asterisks represent significant differences in expressions compared with the control (0 h) based on *t*-test (* *p* < 0.05, ** *p* < 0.01).

The stress-responsive elements detected in *SlBURP* promoters indicated that *SlBURPs* may be related to tomato’s response to abiotic stress (Figure 4). Therefore, we explored the expression dynamics of *SlBURPs* (except *SlUSP4* and *SlUSP5* with undetectable expressions in leaves both under control and treatments) under drought (20% PEG-6000), salt (200 mM and 400 mM) and cold (4 °C) stresses. As shown in Figure 6, the expressions of all eight *SlBURPs* were induced by the drought, salt and cold treatments. Under drought treatment, the expression levels of *SlBNM2* and *SlUSP6* were significantly down-regulated. In contrast, the expression levels of *SlPG2* and *SlPG5* were significantly up-regulated. The remaining *SlBURPs* showed alternately induced expression dynamics (Figure 6A). Under salt treatment, the expressions of *SlRD1*, *SlBNM1*, *SlUSP6* and *SlPG3* were significantly up-regulated at 400 mM or both 200 mM and 400 mM conditions, with *SlRD1* showing the most significant up-regulation (5.72-fold). The expression of *LePG1* was significantly down-regulated at 3 days of treatment. The expressions of *SlBNM2*, *SlPG2* and *SlPG5* were not significantly changed under 200 mM salt treatment but were significantly up-regulated at 12 h and 24 h, and then significantly down-regulated at 3 days under 400 mM salt treatment (Figure 6B). Under cold treatment, the expressions of *SlPG3* and *SlUSP6* were significantly up-regulated, while those of *SlBNM1* and *LePG1* were significantly down-regulated. The expressions of the remaining *SlBURPs* were significantly up-regulated over 6–24 h or at 24 h, and then significantly down-regulated at 48 h (Figure 6C). Overall, the variable induction of *SlBURPs* under the tree treatments indicated their potential roles in response to abiotic stresses.

### 2.7. Expression Analysis of SlBURPs Under ABA Treatment

**Figure 7:**
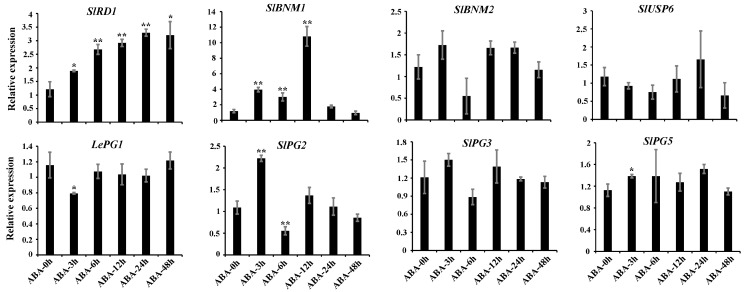
Expressions of 8 *SlBURPs* in tomato leaves after ABA treatment. The asterisks represent significant differences expressions compared with the control (0 h) based on *t*-test (* *p* < 0.05, ** *p* < 0.01).

The expression of eight *SlBURPs* were investigated by qRT-PCR to explore whether *SlBURPs* were responsive to ABA treatment. The result showed that the expressions of 5 *SlBURPs* (except *SlBNM2*, *SlUSP6* and *SlPG3*) were sensitive to ABA treatment (Figure 7). Among them, *SlRD1*, *SlBNM1* and *SlPG5* showed significantly up-regulated expressions, with constant increasing in the expression of *SlRD1* and most significantly up-regulated at 12 h (10.82-fold) in the expression of *SlBNM1*. The expression of *LePG1* were significantly down-regulated at 3 h. The expression of *SlPG2* were significantly up-regulated at 3 h, then down-regulated at 6 h. The result suggested certain *SlBURPs* may be involved in ABA response pathway.

### 2.8. SlRD1 Improved Salt and Drought Tolerance of Transgenic Arabidopsis

**Figure 8:**
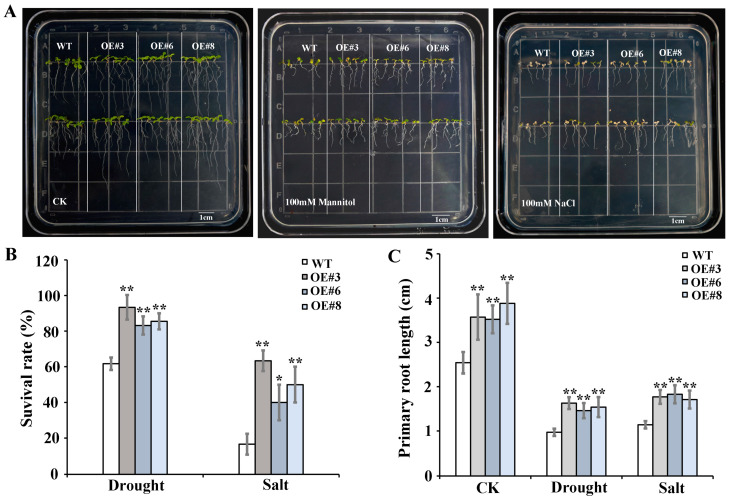
Phenotypic analysis of ectopically expressed *SlRD1 Arabidopsis* lines under drought and salt stresses. (**A**) Phenotypes of transgenic *Arabidopsis* lines and WT under 100 mM mannitol and 100 mM NaCl treatments. (**B**) The survival rates of transgenic *Arabidopsis* lines and WT under mannitol and NaCl treatments. (**C**) The primary root lengths of transgenic *Arabidopsis* lines and WT under control, 100 mM mannitol and 100 mM NaCl treatments. WT, wild type. CK, control. The asterisks represent significant differences compared with the control based on *t*-test (* *p* < 0.05, ** *p* < 0.01).

Based on the phylogenetic analysis (Figure 1), we found that *SlRD1* was most homologous with *AtRD22*, a drought-responsive *BURP* gene [5]. Furthermore, *cis*-acting element analysis (Figure 4) and expression analysis under abiotic stress and ABA treatments (Figure 6 and Figure 7) showed that stress- and ABA-responsive elements were present in the *SlRD1* promoter, and the expression of *SlRD1* was significantly induced by the three abiotic stress and ABA treatments, especially salt and ABA. To further explore the potential function of *SlRD1*, we employed the ectopic expression of *SlRD1* in *Arabidopsis*. Three successful transformations of *SlRD1* into *Arabidopsis* lines (OE#3, OE#6 and OE#8) were verified using qRT-PCR (Figure S2A). The primary roots of 5-day-old transgenic *Arabidopsis* seedlings grown on 1/2 Murashige and Skoog (MS) media were significantly longer than those of wild-type (WT) *Arabidopsis* (Figure S2B,C). Furthermore, after 5 days of 100 mM mannitol and 100 mM NaCl treatments, respectively, the yellowing and wilting of transgenic *Arabidopsis* leaves were less severe compared with the WT leaves (Figure 8A). The survival rates of transgenic *Arabidopsis* plants under drought and salt treatments were both significantly higher than those of the WT (Figure 8B). The primary roots of transgenic *Arabidopsis* plants were significantly longer compared with the WT under the control, drought and salt treatments (Figure 8C). In addition, after 200 mM NaCl treatment (7 days) and drought treatment (3 days and 10 days), transgenic *Arabidopsis* plants showed higher resistances than the WT (Figures S3 and S4). These results indicated that overexpression of *SlRD1* improved drought and salt tolerance in *Arabidopsis*, potentially through promoting root elongation.

### 2.9. SlRD1 Improved ABA Sensitivity of Transgenic Arabidopsis Seeds

**Figure 9:**
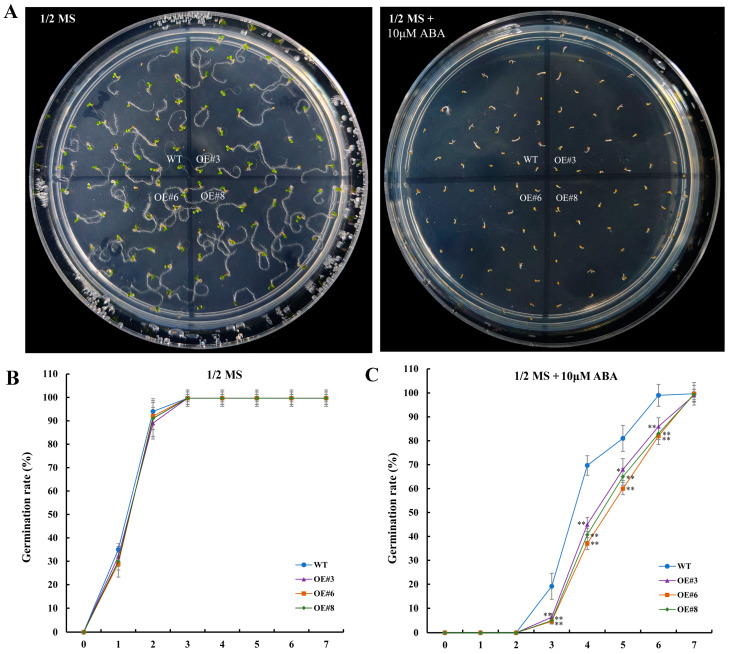
Seed germination of ectopically expressed *SlRD1 Arabidopsis* lines and WT under control and 10 μM ABA treatment. (**A**) Seed germination phenotypes of transgenic *Arabidopsis* lines and WT grown on 1/2 MS media and 1/2 MS media with 10 μM ABA for 5 days. (**B**) The seed germination rates of transgenic *Arabidopsis* lines and WT grown on 1/2 MS media from 0 days to 7 days. (**C**) The seed germination rates of transgenic *Arabidopsis* lines and WT grown on 1/2 MS media with 10 μM ABA from 0 day to 7 days. The asterisks represent significant differences compared with the control based on *t*-test (** *p* < 0.01).

The expression of *SlRD1* was significantly and constantly up-regulated under ABA treatment (Figure 7). In order to investigate whether *SlRD1* was involved in ABA response, the germination rates of transgenic *Arabidopsis* lines and the WT under control and ABA treatments were counted (Figure 9). The germination rates showed no significant difference between transgenic *Arabidopsis* seeds and WT grown on 1/2 MS media from 0 day to 7 days (Figure 9B). However, the germination rates of transgenic *Arabidopsis* seeds and WT were both reduced when grown on 1/2 MS media with 10 μM ABA. Notably, the germination rates of transgenic *Arabidopsis* seeds were significantly lower than the WT from 3 days to 6 days (Figure 9C). The results indicated that the ectopic expression of *SlRD1* in *Arabidopsis* enhanced sensitivity to exogenous ABA.

## 3. Discussion

Many *BURP* genes have been studied for their critical roles in various developmental processes and stress responses [3,5,18,21]. With the broadening of plant genomics publications and research, the genome-wide identification and analysis of *BURP* gene family also have been studied in many plants. In our study, the phylogenetic analysis of BURP members revealed that eight subfamilies were classified, with fourteen tomato BURP members distributed in BNM2-like, USP-like, RD22-like and PG1β-like subfamilies (Figure 1). The similarity of gene structure and conserved motif arrangement of *SlBURPs* within same subfamily supported the classification of subfamilies. The various numbers of exons and motifs of *SlBURPs* from different subfamilies may be related to the functional diversity. The similar results of gene structures were found in cotton [6], rose [10] and legumes [25]. Also, the phylogenetic results in BURPs of cotton [6], soybean [26] and alfalfa (*Medicago truncatula*) [7] were found. Gene duplication events and uneven distribution of chromosomes (Figure 2) have been observed in the tomato *BURP* family indicating that this family may have an expansion and functional redundancy, similar to findings in cotton [6] and alfalfa [7].

The roles of *BURP* family genes in plant-specific developmental processes, including seeds [17], fruit [19,20] and flowers [14,15], have been widely reported. The expression patterns of *SlBURPs* in different tomato tissues were investigated to assess the potential functions in tomato development (Figure 5). The expressions of some *SlBURPs* showed distinct tissue-specificity. For instance, *SlBNM1* and *SlBNM2* were more prevalent in roots than other tissues, similar to the homologous *AtUSP1* [5]. *SlUSP5* and *SlUSP6* showed root-specific expressions, indicating the potential roles of these genes in root development. The fruit specific expression (especially in tomato fruit ripening) of *LePG1* has been studied [20] and were also proven in our study. Although belonging to the PG1β-like subfamily, *SlPG3* showed a similar expression pattern to *LePG1*, while *SlPG2* and *SlPG5* exhibited different expression patterns, with high expression in the stem. Similarly, the conservation and divergence of *BURP* expressions have also been found in jujube [8] and soybean [26]. In addition, *SlRD1* exhibited high expression in leaf, similar to *AtRD1* [5], and also has the highest expression in fruit, suggesting a variety of biological functions during plant evolution.

The stress response elements were widely present in *SlBURP* promoters (Figure 4), similar to previous studies [6,7,8], indicating the possible response to various stresses. The result of qRT-PCR analysis showed that the expressions of all *SlBURPs* could be induced by drought, salt, and cold treatments, although with different levels of induction and trends (Figure 6). Under drought treatment, the expression of *SlPG3* showed the most significant up-regulation at 24 h, followed by *SlPG2* at 24 h and *SlRD1* at 1 h, which were similar to *PvBURP4*/*8*, *MtBURP30*/*31*/*32* (belonging to PG1β-like subfamilies), *PvBURP3* and *MtBURP10*/*11* (belonging to RD22-like subfamilies) expression in *Phaseolus vulgaris* [9] and alfalfa [7]. In addition, *SlUSP6* showed strikingly down-regulated expression, indicating this gene may have a negative response to drought stress. Under salt treatment, the expressions of most *SlBURPs*, except *LePG1*, were up-regulated at 12 h and 24 h, similarly to the expressions of *ZjBURPs* and *GhBURPs* under salt stress [6,8]. Under cold treatment, the expressions of most *SlBURPs*, except *LePG1* and *SlBNM1*, were significantly up-regulated at 24 h, which was consistent with expressions of *CaBURP* in *Cicer arietinum* [25], whereas the opposite was shown for *ZjBURPs* [8]. This indicated that *BURP* genes of different species may play various roles in plant response to cold. ABA showed an important role in plant response to abiotic stress [27]. Five *SlBURPs*, specifically *SlRD1* and *SlBNM1* with the most significantly up-regulated expressions at different points, showed various responses to ABA treatment (Figure 7) and similar expressions were observed for the *PvBURP* family [9] and *ZmRD22B* [28].

The identification and expressions of *BURP* genes have been performed in many plants, but their largely unknown functions provide limited understanding of this gene family. Based on the homologous stress-related *AtRD22* and its high response to multiple abiotic stresses and ABA treatment, *SlRD1* was chosen to be ectopically expressed in *Arabidopsis* to further explore the role of this gene in response to stress. Higher resistance was detected in transgenic *Arabidopsis* plants than WT indicating that *SlRD1* may positively regulate the response to drought and salt stresses (Figure 8, Figures S3 and S4). In *Arabidopsis*, *AtRD22* was involved in the drought stress response [5]. Ectopic expression of *BnBDC1*, homologous to *AtRD22*, enhanced the drought and cold resistance of *Arabidopsis* [29]. Similarly, ectopic expression of *GmRD22* improved the resistance of *Arabidopsis* and rice to salt stress [22]. The ectopic expression of *RvBURP4* in *Arabidopsis* suggested the diverse functions of this gene in response to salt and drought stresses [10]. These results suggest that *BURPs*, especially members of the RD22-like subfamily, played critical roles in the plant’s abiotic stress response. In addition, the higher sensitivity of transgenic *Arabidopsis* seeds to ABA treatment than those of the WT indicated that *SlRD1* may be involved in the ABA signaling pathway (Figure 9). This result was supported by the four ABA response elements (AREB) in *SlRD1* promoter (Figure 4) and the significantly up-regulated expression of *SlRD1* under ABA treatment at all treatment points (Figure 7). *AtRD22* was part of the ABA-mediated drought stress response [5]. This is consistent with another study that reported that the *RcBURP4* transgenic *Arabidopsis* was more susceptible to ABA treatment than the WT during seed germination [10].

## 4. Materials and Methods

### 4.1. SlBURP Family Member Identification in Tomato

The genome sequences of tomato (version ITAG4.0) and BURP domain model file (PF03181) were obtained from Sol Genomics Network (http://solgenomics.net/ (accessed on 19 September 2023)) and the Pfam database (https://pfam.xfam.org/ (accessed on 19 September 2023) [30], respectively. The candidate *SlBURP* genes from tomato genome were screened using HMMER 3.0 with the threshold set as E-value < 1 × 10^−10^ [6]. The NCBI Conserved Domain Database (https://www.ncbi.nlm.nih.gov/Structure/cdd/wrpsb.cgi (accessed on 19 September 2023)) and SMART (http://smart.embl-heidelberg.de/ (accessed on 19 September 2023)) were used to individually identify the BURP domain in SlBURP protein sequences [31]. The information of SlBURP proteins, including molecular weights (Mw), isoelectric points (pI) and grand average of hydropathicity, was predicted using the ExPASy online tool (http://web.expasy.org/protparam/ (accessed on 19 September 2023)) [32].

### 4.2. SlBURP Phylogeny, Chromosomal Location and Duplication Analysis

The BURP protein sequences of *Arabidopsis*, rice, maize, cotton and two host BURP proteins (BNM2 and VfUSP) were downloaded from TAIR (https://www.arabidopsis.org/ (accessed on 22 September 2023)), Phytozome 13 (https://phytozome-next.jgi.doe.gov/ (accessed on 22 September 2023)) and NCBI ((www.ncbi.nlm.nih.gov/protein (accessed on 22 September 2023)). The phylogenetic trees were established using the Neighbor-Joining method in MEGA 7.0, and the bootstrap replications was set to 1000 [33].

The chromosomal location of *SlBURPs* was generated using TBtools, based on the genomic location information from the gff3 file of the tomato genome [34]. Multiple sequence alignment of *SlBURPs* was performed using Clustal Omega on the EMBL-EBI website (https://www.ebi.ac.uk/Tools/msa/clustalo/ (accessed on 9 January 2024)). The segmental duplication genes were confirmed if the alignment coverage and the sequence similarities were ≥80%. Moreover, if the physical locations of the duplicated genes on the same chromosome were ≤200 kb apart, these genes were regarded as tandem duplication clusters [35].

### 4.3. SlBURP Gene Structures and Conserved Motifs

The gene structure image of *SlBURPs* was drawn using GSDS 2.0 (http://gsds.cbi.pku.edu.cn/ (accessed on 10 January 2024), based on the intron-exon position from the gff3 file of the tomato genome. The conserved BURP domain and amino acid sites of SlBURP proteins were analyzed using DNAMAN 8.0. SignalP 4.0 Server (http://www.cbs.dtu.dk/services/SignalP/ (accessed on 10 January 2024)) was employed to predicate the signal peptides in SlBURP proteins [36]. The conserved motifs of SlBURP proteins were predicted using the MEME online tool (https://meme-suite.org/meme/tools/meme (accessed on 10 January 2024)) with 10 motifs as maximum number.

### 4.4. Cis-Acting Elements in SlBURP Promoters

The 1500 bp upstream sequences from the start codon of *SlBURPs* were extracted from the tomato genome via TBtools [34]. Then, the PlantCARE website (http://bioinformatics.psb.ugent.be/webtools/plantcare/html/ (accessed on 21 September 2023)) was employed to investigated potential *cis*-acting elements in *SlBURP* promoters [37].

### 4.5. Plant Growth and Treatments

The tomato cv. ‘Micro-Tom’ was sown and transplanted in a growth chamber under light conditions of 26 °C for 16 h and dark conditions of 18 °C for 8 h. The different tomato tissues were collected and frozen in liquid nitrogen, and then stored at −80 °C. The six-leaf-stage tomato seedlings were used for the abiotic stress and ABA treatments. For drought and salt treatments, the tomato seedlings were irrigated with 20% (*w*/*v*) PEG6000 or 200 mM NaCl or 400 mM NaCl. The leaf samples were collected at 0, 1, 3, 6, 12, and 24 h for PEG6000 treatment and 0, 12, 24 h and 3 days for NaCl treatment. For cold treatment, the tomato seedlings were transferred to an artificial climate chamber set at 4 °C. The leaf samples were collected at 0, 6, 12, 24, and 48 h. For ABA treatment, the leaves of the tomato seedlings were sprayed with 100 μM ABA and collected at 0, 3, 6, 12, 24, and 48 h. Each time point of treatment contained tree biological replicates with five tomato seedlings. The collected leaf samples were rapidly frozen in liquid nitrogen and stored at −80 °C for further analysis.

### 4.6. RNA Extraction and qRT-PCR Analysis

The Polysaccharide Polyphenol Plant RNA Extraction kit (Tiangen, Beijing, China) and StarScript II First-strand cDNA Synthesis Mix with gDNA Remover (GenStar, Beijing, China) were chosen for RNA extraction and cDNA synthesis, respectively. RealStar Green Fast Mixture with ROX (2×) (GenStar, Beijing, China) was used for qRT-PCR via an Applied Biosystems StepOnePlus instrument. The relative expressions of *SlBURPs* were calculated using the 2^−ΔΔCT^ method. And, *SlEF1α* and *Actin* were used as the internal reference genes in tomato and *Arabidopsis*, respectively. Then, the *t*-test was used to analyze the significance of differences in *SlBURP* expressions. The specific primers of *SlBURPs* were designed using Primer 5.0 and were listed in Table S2.

### 4.7. Generation of SlRD1 Transgenic Arabidopsis Plants

The CDS of *SlRD1* was amplificated, and then the correct sequence was cloned into the overexpression vector (pBI121). The recombinant plasmid, 35S::*SlRD1*, was obtained and transformed into an *Agrobacterium* strain (GV3101). The floral dripping method was used to transform the *Arabidopsis* cv. ‘Col-0’ [38]. The T_0_ transgenic *Arabidopsis* seeds were screened on 1/2 MS media with 15 µg/L. The *SlRD1* expressions of T_3_ homozygous transgenic *Arabidopsis* seedings were detected using qRT-PCR. The primers used were listed in Table S2.

### 4.8. Stress Treatments of SlRD1 Transgenic Arabidopsis Plants

The WT and transgenic *Arabidopsis* seeds were evenly sown on 1/2 MS media and grown normally for 5 days in a plant growth chamber under light conditions at 22 °C for 16 h and dark for 8 h. The lengths of primary roots were measured and statistically analyzed with three biological replicates. For the stress treatment assays, 3-day-old WT and transgenic *Arabidopsis* seedings were transferred to 1/2 MS, 1/2 MS with 100 mM mannitol and 100 mM NaCl media, respectively. The lengths of the primary roots and survival rates were calculated and statistically analyzed after 5 days under normal, drought and salt conditions with three biological replicates. Four-week-old WT and transgenic *Arabidopsis* seedings grown in soil were chosen to 200 mM NaCl and drought treatments, respectively.

For the *Arabidopsis* seed germination assays, the WT and transgenic *Arabidopsis* seeds were sterilized and then evenly sown on 1/2 MS and 1/2 MS with 10 µM ABA media, respectively. Then, the seed germination rates were calculated and statistically analyzed with three biological replicates from days 0 to 7.

## 5. Conclusions

A total of fourteen *SlBURP* genes were confirmed in the tomato genome and classified into BNM2-like, USP-like, RD22-like and PG1β-like subfamilies. The closely related *SlBURPs* contained similar gene structures and motif arrangements. Tandem duplication may be the main cause of tomato *BURP* gene family expansion. The expressions of *SlBURPs* in different tomato tissues were revealed to be spatio-temporally specific. The analyses of *cis*-acting elements and expression dynamics under abiotic stress and ABA treatments implied that *SlBURPs* may play important roles in response to various stresses. Moreover, the ectopic expression of *SlRD1* in *Arabidopsis* facilitated the elongation of primary roots under normal, drought and salt conditions. The tolerance of *SlRD1* transgenic *Arabidopsis* under drought and salt treatments was higher compared with the WT, suggesting a positive role for this gene in response to drought and salt stresses. In summary, our study deepens the understanding of the *BURP* gene family and provides a foundation for further exploration of the potential functions of tomato *BURP* genes.

## Data Availability

Data are contained within the article and Supplementary Materials.

## Supplementary Materials

The following supporting information can be downloaded at: https://www.mdpi.com/article/10.3390/ijms252312539/s1.

## References

[1] Albacete A., Ghanem M.E., Martínez-Andújar C., Acosta M., Sánchez-Bravo J., Martínez V., Lutts S., Dodd I.C., Pérez-Alfocea F. (2008). Hormonal changes in relation to biomass partitioning and shoot growth impairment in salinized tomato (*Solanum lycopersicum* L.) plants. J Exp. Bot..

[2] Liang Y., Ma F., Li B., Guo C., Hu T., Zhang M., Liang Y., Zhu J., Zhan X. (2022). A bHLH transcription factor, SlbHLH96, promotes drought tolerance in tomato. Hortic. Res..

[3] Yu S., Yang F., Zou Y., Yang Y., Li T., Chen S., Wang Y., Xu K., Xia H., Luo L. (2022). Overexpressing *PpBURP2* in rice increases plant defense to abiotic stress and bacterial leaf blight. Front. Plant. Sci..

[4] Liu H., Ma Y., Chen N., Guo S., Liu H., Guo X., Chong K., Xu Y. (2014). Overexpression of stress-inducible OsBURP16, the β subunit of polygalacturonase 1, decreases pectin content and cell adhesion and increases abiotic stress sensitivity in rice. Plant Cell Environ..

[5] Harshavardhan V.T., Van Son L., Seiler C., Junker A., Weigelt-Fischer K., Klukas C., Altmann T., Sreenivasulu N., Bäumlein H., Kuhlmann M. (2014). AtRD22 and AtUSPL1, members of the plant-specific BURP domain family involved in *Arabidopsis thaliana* drought tolerance. PLoS ONE.

[6] Sun H., Wei H., Wang H., Hao P., Gu L., Liu G., Ma L., Su Z., Yu S. (2019). Genome-wide identification and expression analysis of the BURP domain-containing genes in *Gossypium hirsutum*. BMC Genom..

[7] Li Y., Chen X., Chen Z., Cai R., Zhang H., Xiang Y. (2016). Identification and expression analysis of BURP domain-containing genes in *Medicago truncatula*. Front. Plant Sci..

[8] Wang W., Zhang Z., Li X. (2022). Identification and expression analysis of BURP domain-containing genes in jujube and their involvement in low temperature and drought response. BMC Genom..

[9] Kavas M., Yıldırım K., Seçgin Z., Abdulla M.F., Gökdemir G. (2021). Genome-wide identification of the BURP domain-containing genes in *Phaseolus vulgaris*. Physiol. Mol. Biol. Plants..

[10] Fu L., Zhang Z., Wang H., Zhao X., Su L., Geng L., Lu Y., Tong B., Liu Q., Jiang X. (2022). Genome-wide analysis of BURP genes and identification of a BURP-V gene *RcBURP4* in *Rosa chinensis*. Plant Cell Rep..

[11] Teerawanichpan P., Xia Q., Caldwell S.J., Datla R., Selvaraj G. (2009). Protein storage vacuoles of Brassica napus zygotic embryos accumulate a BURP domain protein and perturbation of its production distorts the PSV. Plant Mol. Biol..

[12] Chesnokov Y.V., Meister A., Manteuffel R. (2002). A chimeric green fluorescent protein gene as an embryogenic marker in transgenic cell culture of *Nicotiana plumbaginifolia* Viv. Plant Sci..

[13] Chen L., Miyazaki C., Kojimai A., Saito A., Adachi T. (1999). Isolation and characterization of a gene expressed during early embryo sac development in apomictic guinea grass (*Panicum maximum*). J. Plant Physiol..

[14] Wang A., Xia Q., Xie W., Datla R., Selvaraj G. (2003). The classical Ubisch bodies carry a sporophytically produced structural protein (RAFTIN) that is essential for pollen development. Proc. Natl. Acad. Sci. USA.

[15] Jeon J.S., Chung Y.Y., Lee S., Yi G.H., Oh B.G., An G. (1999). Isolation and characterization of an anther-specific gene, RA8, from rice (*Oryza sativa* L.). Plant Mol. Biol..

[16] Van Son L., Tiedemann J., Rutten T., Hillmer S., Hinz G., Zank T., Manteuffel R., Bäumlein H. (2009). The BURP domain protein AtUSPL1 of *Arabidopsis thaliana* is destined to the protein storage vacuoles and overexpression of the cognate gene distorts seed development. Plant Mol. Biol..

[17] Batchelor A.K., Boutilier K., Miller S.S., Hattori J., Bowman L.A., Hu M., Lantin S., Johnson D.A., Miki B.L. (2002). *SCB1*, a BURP-domain protein gene, from developing soybean seed coats. Planta.

[18] Shan C.M., Shangguan X.X., Zhao B., Zhang X.F., Chao L.M., Yang C.Q., Wang L.J., Zhu H.Y., Zeng Y.D., Guo W.Z. (2014). Control of cotton fibre elongation by a homeodomain transcription factor GhHOX3. Nat. Commun..

[19] Watson C.F., Zheng L., DellaPenna D. (1994). Reduction of tomato polygalacturonase beta subunit expression affects pectin solubilization and degradation during fruit ripening. Plant Cell.

[20] Zheng L., Heupel R.C., DellaPenna D. (1992). The beta subunit of tomato fruit polygalacturonase isoenzyme 1: Isolation, characterization, and identification of unique structural features. Plant Cell.

[21] Dong Q., Tian Y., Zhang X., Duan D., Zhang H., Yang K., Jia P., Luan H., Guo S., Qi G. (2024). Overexpression of the transcription factor MdWRKY115 improves drought and osmotic stress tolerance by directly binding to the *MdRD22* promoter in apple. Hortic. Plant J..

[22] Wang H., Zhou L., Fu Y.P., Cheung M.Y., Wong F.L., Phang T.H., Sun Z.X., Lam H.M. (2012). Expression of an apoplast-localized BURP-domain protein from soybean (GmRD22) enhances tolerance towards abiotic stress. Plant Cell Environ..

[23] Banzai T., Sumiya K., Hanagata N., Dubinsky Z., Karube I. (2002). Molecular cloning and characterization of genes encoding BURP domain-containing protein in the mangrove, *Bruguiera gymnorrhiza*. Trees.

[24] Tang Y., Cao Y., Gao Z., Ou Z., Wang Y., Qiu J., Zheng Y. (2014). Expression of a vacuole-localized BURP-domain protein from soybean (SALI3-2) enhances tolerance to cadmium and copper stresses. PLoS ONE.

[25] Chitkara P., Poddar N., Singh A., Kumar S. (2022). BURP domain-containing genes in legumes: Genome-wide identification, structure, and expression analysis under stresses and development. Plant Biotechnol. Rep..

[26] Xu H., Li Y., Yan Y., Wang K., Gao Y., Hu Y. (2010). Genome-scale identification of soybean BURP domain-containing genes and their expression under stress treatments. BMC Plant Biol..

[27] Verma V., Ravindran P., Kumar P.P. (2016). Plant hormone-mediated regulation of stress responses. BMC Plant Biol..

[28] Phillips K., Ludidi N. (2017). Drought and exogenous abscisic acid alter hydrogen peroxide accumulation and differentially regulate the expression of two maize RD22-like genes. Sci. Rep..

[29] Yu S., Zhang L., Zuo K., Li Z., Tang K. (2004). Isolation and characterization of a BURP domain-containing gene *BnBDC1* from *Brassica napus* involved in abiotic and biotic stress. Physiol. Plant..

[30] Mistry J., Chuguransky S., Williams L., Qureshi M., Salazar G.A., Sonnhammer E.L.L., Tosatto S.C.E., Paladin L., Raj S., Richardson L.J. (2021). Pfam: The protein families database in 2021. Nucleic. Acids Res..

[31] Zhao Y., Sun X., Zhou J., Liu L., Huang L., Hu Q. (2024). Identification and functional exploration of *BraGASA* genes reveal their potential roles in drought stress tolerance and sexual reproduction in *Brassica rapa* L. ssp. pekinensis. Int. J. Mol. Sci..

[32] Wilkins M.R., Gasteiger E., Bairoch A., Sanchez J.C., Williams K.L., Appel R.D., Hochstrasser D.F. (1999). Protein identification and analysis tools in the ExPASy server. Methods Mol. Biol..

[33] Kumar S., Stecher G., Tamura K. (2016). MEGA7: Molecular evolutionary genetics analysis version 7.0 for bigger datasets. Mol. Biol. Evol..

[34] Chen C., Chen H., Zhang Y., Thomas H.R., Frank M.H., He Y., Xia R. (2020). TBtools: An integrative toolkit developed for interactive analyses of big biological data. Mol. Plant..

[35] Fu Y., Cheng M., Li M., Guo X., Wu Y., Wang J. (2020). Identification and characterization of PLATZ transcription factors in wheat. Int. J. Mol. Sci..

[36] Petersen T.N., Brunak S., von Heijne G., Nielsen H. (2011). SignalP 4.0: Discriminating signal peptides from transmembrane regions. Nat. Methods..

[37] Lescot M., Déhais P., Thijs G., Marchal K., Moreau Y., Van de Peer Y., Rouzé P., Rombauts S. (2002). PlantCARE, a database of plant cis-acting regulatory elements and a portal to tools for in silico analysis of promoter sequences. Nucleic. Acids Res..

[38] Zhang X., Henriques R., Lin S.S., Niu Q.W., Chua N.H. (2006). *Agrobacterium*-mediated transformation of *Arabidopsis thaliana* using the floral dip method. Nat. Protoc..

