# Supplementary material for: Genome-Wide Analysis of BURP Domain-Containing Gene Family in Solanum lycopersicum and Functional Analysis of SlRD1 Under Drought and Salt Stresses
Source: Int J Mol Sci. 2024 Nov 22;25(23):12539. doi: 10.3390/ijms252312539 (PMC11641390; doi:10.3390/ijms252312539)
Supplement: Supplementary file 1 [file ijms-25-12539-s001.zip › ijms-3299074-supplementary.pdf]

Supplementary Materials

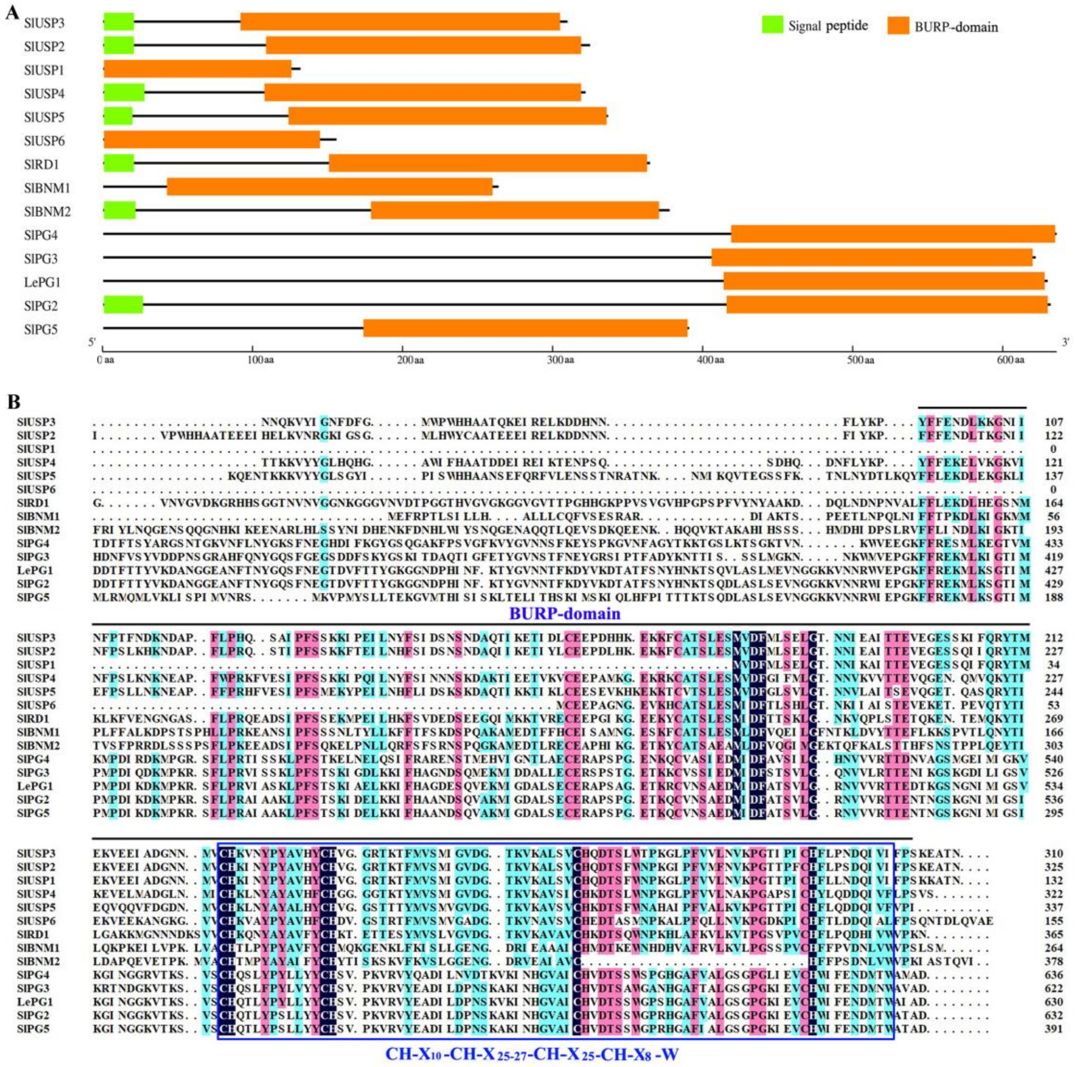

Figure S1. The conserved domain **(A)** and multiple sequence alignment **(B)** analysis of SlBURP family proteins. The black line represents the BURP domain. Blue box represents highly conserved motif in BURP domain.

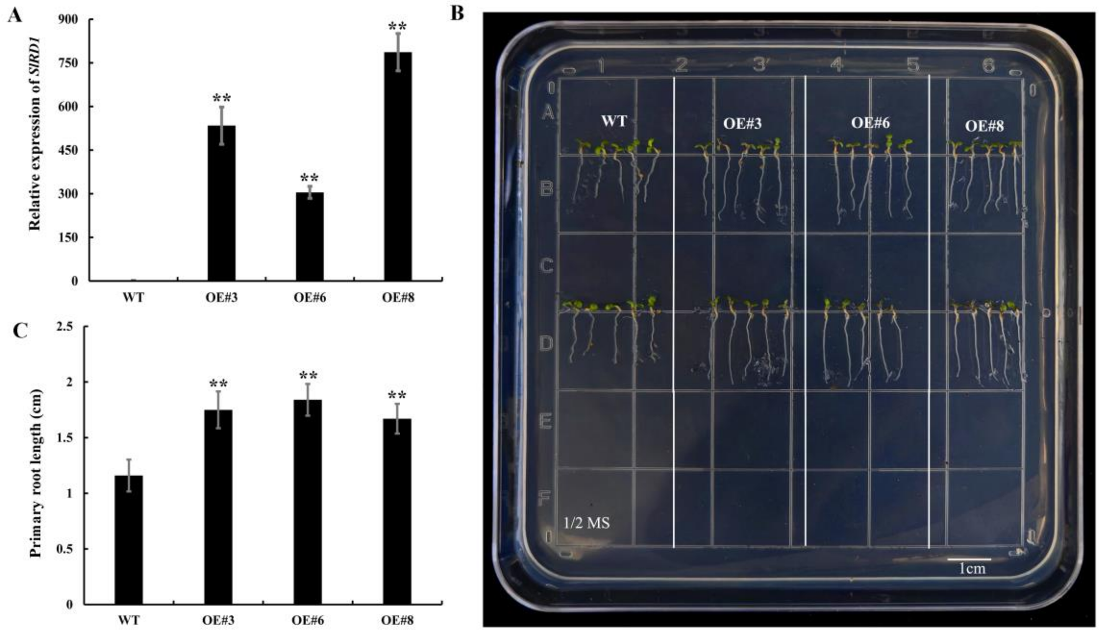

Figure S2. Phenotypic analysis of *SlRD1* transgenic *Arabidopsis* lines (OE#3, OE#6 and OE#8). **(A)** The expressions of *SlRD1* in the WT and transgenic *Arabidopsis* seedings. **(B)** The primary roots of the WT and transgenic *Arabidopsis* seedings on 1/2 MS medium for 5 days. **(C)** The primary root lengths of the WT and transgenic *Arabidopsis* lines on 1/2 MS medium for 5 days. WT, wild type. The asterisks represent significant differences compared with the control based on *t*-test (** $p < 0.01$).

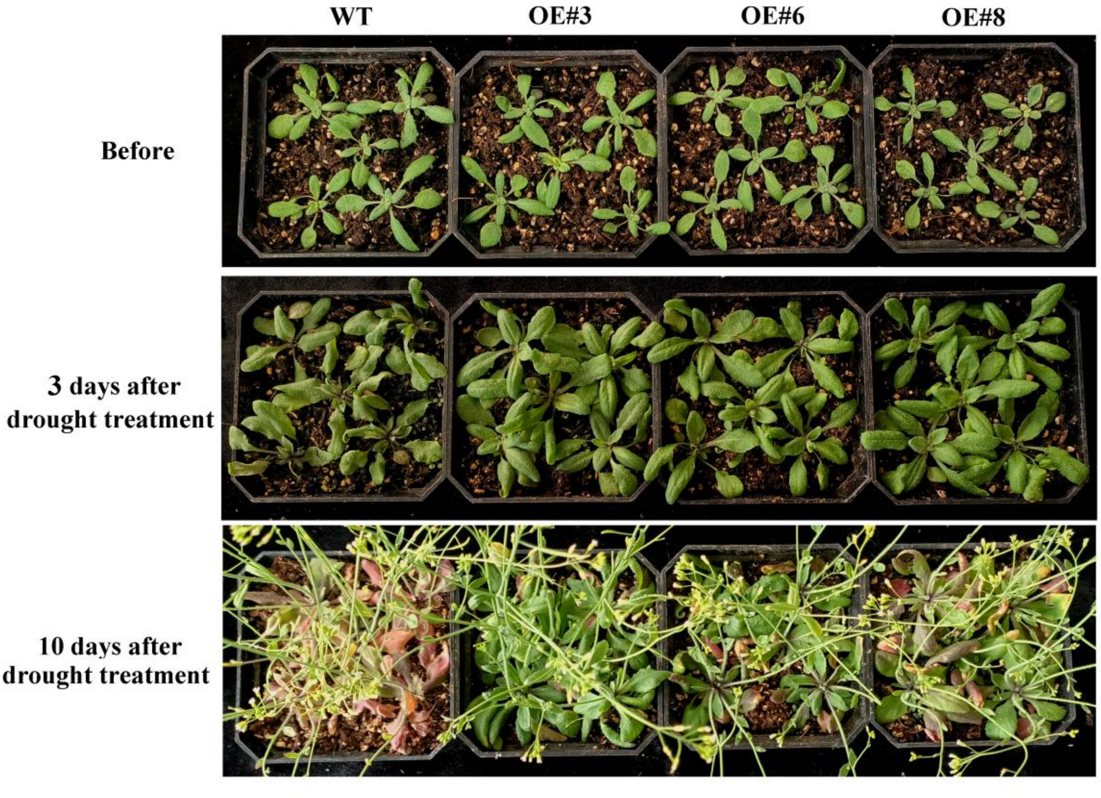

Figure S3. Phenotypes of transgenic *Arabidopsis* lines and the WT under drought treatment.

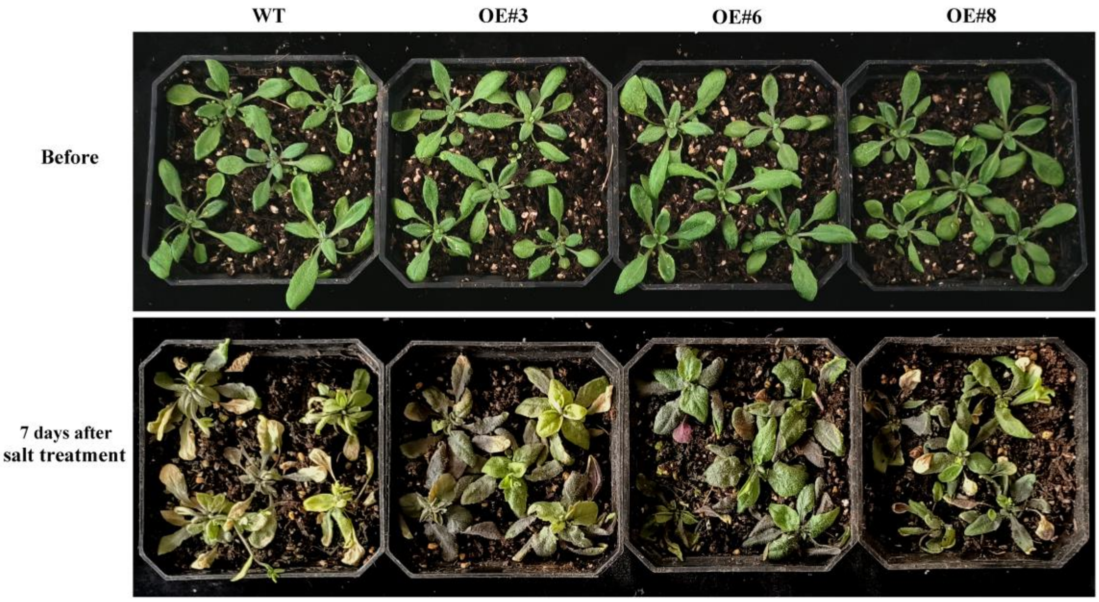

Figure S4. Phenotypes of transgenic *Arabidopsis* lines and the WT under 200 mM NaCl treatment.

Table S1. Physicochemical properties of *BURP* gene family in tomato

Gene	Gene ID	Location in chromosome	Length of amino acids (aa)	Molecular weight (kDa)	pI	Grand average of hydropathicity (GRAVY)	Exon number	Location of BURP-domain
SlBNM1	Solyc01g109470.3.1	SL4.0ch01:88755996:88757409	264	30.03	7.1	-0.031	2	43-260
SlBNM2	Solyc01g109500.4.1	SL4.0ch01:88770815:88772478	378	43.05	6.19	-0.593	3	179-371
SlUSP1	Solyc02g062310.1.1	SL4.0ch02:31903164:31903562	132	14.76	6.95	-0.062	1	1-126
SlUSP3	Solyc02g062313.1.1	SL4.0ch02:31908043:31909432	310	35.47	6.22	-0.307	3	92-305
SlUSP2	Solyc02g062320.3.1	SL4.0ch02:31944540:31945829	325	37.22	6.05	-0.303	3	109-319
SlUSP4	Solyc02g062300.3.1	SL4.0ch02:31883883:31886335	322	36.32	8.54	-0.248	3	108-319
SlUSP5	Solyc05g015300.4.1	SL4.0ch05:10345166:10347805	337	38.17	7.65	-0.217	3	124-336
SlUSP6	Solyc08g068130.1.1	SL4.0ch08:55314884:55315354	156	16.98	5.13	-0.190	1	1-145
LePG1	Solyc05g005560.4.1	SL4.0ch05:448750:451355	630	68.96	6.96	-0.539	2	414-628
SlPG2	Solyc05g005540.4.1	SL4.0ch05:426180:429075	632	69.11	6.19	-0.609	2	416-630
SlPG3	Solyc05g005570.3.1	SL4.0ch05:453888:457026	622	68.70	8.49	-0.498	1	406-620
SlPG4	Solyc03g114240.4.1	SL4.0ch03:58696278:58698568	636	69.47	8.96	-0.427	3	419-635
SlPG5	Solyc05g005550.4.1	SL4.0ch05:430763:433493	391	43.45	9.71	0.017	2	174-390
SlRD1	Solyc08g068150.4.1	SL4.0ch08:55336787:55339646	365	39.55	7.26	-0.438	4	151-363

Table S2. The primers used in this study

Primer name	Primer sequence (5'-3')	Purpose
SlBNM1-Q-F	TGAGTGAAAGCAGAGCCAGAG	qRT-PCR
SlBNM1-Q-R	GAGGAGAAGTTGAAGGGTCTTTT	qRT-PCR
SlBNM2-Q-F	TCTCAGCAGGGCAATCACAA	qRT-PCR
SlBNM2-Q-R	TGTTTGTCGCTCACTTCTTGT	qRT-PCR
LePG1-Q-F	TGTTGTAGGTGGAGATGGTGA	qRT-PCR
LePG1-Q-R	TGTGCAGCATTCAATGGAGATG	qRT-PCR
SlPG2-Q-F	TGCATTCACTACACACCTCCA	qRT-PCR
SlPG2-Q-R	AGAGTTGAATCCGAGACCAGA	qRT-PCR
SlPG3-Q-F	TGGTCAAAATGCAAATGGGGAG	qRT-PCR
SlPG3-Q-R	CATTACCTCCAACGCCGTAGT	qRT-PCR
SlPG4-Q-F	CACAGGCCTTGCTGCTTTTG	qRT-PCR
SlPG4-Q-R	GCTTCCTACCAACTCCCTCAG	qRT-PCR
SlPG5-Q-F	TCTGCTCCTCTGCCAAATTGT	qRT-PCR
SlPG5-Q-R	AGCCGTCCAGATCCATAGGTA	qRT-PCR
SlRD1-Q-F	GCAATCAAAGAATCTCTCCAGCC	qRT-PCR
SlRD1-Q-R	GTTCACGTTTGTTCCACCGG	qRT-PCR
SlUSP4-Q-F	GCAGCTACTCCACTCTACCATTT	qRT-PCR
SlUSP4-Q-R	TCTTCGTAGTGCCAAGAATGTCA	qRT-PCR
SlUSP5-Q-F	ATGCAGCGATACCTCCAGAG	qRT-PCR
SlUSP5-Q-R	TGTTTTGGCTCAGGAACAACA	qRT-PCR
SlUSP6-Q-F	TCACCTTATCTCACCTGGGAAC	qRT-PCR
SlUSP6-Q-R	GTGACATACAACACCTTTGCCA	qRT-PCR
SlEF1α-Q-F	GACAGGCGTTCAGGTAAGG	qRT-PCR
SlEF1α-Q-R	CCAATGGAGGGTATTCAGC	qRT-PCR
Atactin-Q-F	GCACCCTGTTCTTCTTACCG	qRT-PCR
Atactin-Q-R	AACCCTCGTAGATTGGCACA	qRT-PCR
SlRD1-OE-F	CACGGGGGACTCTAGAATGGAGTTGAAGTTTCTTCACAT	Overexpression vector
SlRD1-OE-R	GACGGCCAGTGAGCTCCTAGTTCTTAGGGACCCAAACA	Overexpression vector
